# Supplementary material for: Neurofeedback training with a low-priced EEG device leads to faster alpha enhancement but shows no effect on cognitive performance: A single-blind, sham-feedback study
Source: PLoS One. 2019 Sep 4;14(9):e0211668. doi: 10.1371/journal.pone.0211668 (PMC6726238; doi:10.1371/journal.pone.0211668)
Supplement: S1 Supporting Information — Original questionnaire. (PDF) [file pone.0211668.s002.pdf]

Questionnaire about Physical Activity, Substance Intake and Sleep

1. Wie gut hast Du gestern Nacht geschlafen?

○ sehr schlecht ○ schlecht ○ eher schlecht ○ weder noch ○ eher gut ○ gut ○ sehr gut

2. Wie gut hast Du gestern Nacht Vergleich zu einer für Dich normalen Nacht geschlafen?

○ viel schlechter als sonst ○ schlechter als als sonst ○ etwas schlechter als sonst ○ wie sonst auch ○ etwas besser als sonst ○ besser als sonst ○ viel besser als sonst

3. Wie gut konntest Du gestern Abend einschlafen?

○ Sehr schlecht ○ schlecht ○ eher schlecht ○ weder noch ○ eher gut ○ gut ○ sehr gut

4. Wie gut konntest Du gestern Abend im Vergleich zu einer für Dich normalen Nacht einschlafen?

○ viel schlechter als sonst ○ schlechter als sonst ○ etwas schlechter als sonst ○ wie sonst auch ○ etwas besser als sonst ○ besser als sonst ○ viel besser als sonst

5. Hast Du heute koffeinhaltige Lebensmittel zu Dir genommen?

Folgende Mengen entsprechen einer Einheit Koffein: Eine Tasse Kaffee, ein Espresso, zwei Tassen Schwarz- oder Grüntee, eine halbe Tafel Zartbitterschokolade, 250 ml Energy Drink, 500 ml Cola.

○ nichts oder fast nichts ○ ca. 0,5 Einheiten ○ ca. 1 Einheit ○ ca. 1,5 Einheiten ○ ca. 2 Einheiten ○ ca. 2,5 Einheiten ○ > 2,5 Einheiten

6. An einem durchschnittlichen Tag, wie viel Koffein hättest du bis zum jetzigen Zeitpunkt zu Dir genommen?

Folgende Mengen entsprechen einer Einheit Koffein: Eine Tasse Kaffee, ein Espresso, zwei Tassen Schwarz- oder Grüntee, eine halbe Tafel Zartbitterschokolade, 250 ml Energy Drink, 500 ml Cola.

○ nichts oder fast nichts ○ ca. 0,5 Einheiten ○ ca. 1 Einheit ○ ca. 1,5 Einheiten ○ ca. 2 Einheiten ○ ca. 2,5 Einheiten ○ > 2,5 Einheiten

7. Wenn ja, wann hast Du heute Koffein zu Dir genommen?

- ☐ zwischen 04:00 und 05:00 Uhr
- ☐ zwischen 05:00 und 06:00 Uhr
- ☐ zwischen 06:00 und 07:00 Uhr
- ☐ zwischen 07:00 und 08:00 Uhr
- ☐ zwischen 08:00 und 09:00 Uhr
- ☐ zwischen 09:00 und 10:00 Uhr
- ☐ zwischen 10:00 und 11:00 Uhr
- ☐ zwischen 11:00 und 12:00 Uhr
- ☐ zwischen 12:00 und 13:00 Uhr
- ☐ zwischen 13:00 und 14:00 Uhr
- ☐ zwischen 14:00 und 15:00 Uhr
- ☐ zwischen 15:00 und 16:00 Uhr
- ☐ zwischen 16:00 und 17:00 Uhr
- ☐ zwischen 17:00 und 18:00 Uhr
- ☐ zwischen 18:00 und 19:00 Uhr
- ☐ zwischen 19:00 und 20:00 Uhr

8. Hast Du Dich gestern körperlich betätigt?
(z.B. Sport getrieben, mit dem Fahrrad gefahren, im Garten gearbeitet, körperlich gearbeitet)

○	○	○	○	○	○	○
gar nicht			mittel			sehr viel

9. Wenn ja, wie lange hast Du Dich gestern sportlich betätigt? (in min)

10. Hast Du Dich heute schon körperlich betätigt?
(z.B. Sport getrieben, mit dem Fahrrad gefahren, im Garten gearbeitet, körperlich gearbeitet)

○	○	○	○	○	○	○
gar nicht			mittel			sehr viel

11. Wenn ja, wie lange hast Du Dich heute sportlich betätigt? (in min)

12. Wie lange befasst Du Dich an einem durchschnittlichen Tag mit körperlichen Tätigkeiten?
(in min.)

13. Hast Du gestern Alkohol konsumiert?
(Die folgenden Mengen entsprechen einer Alkoholeinheit: 0,5 l Bier, 0,2 l Wein, 8 cl Wodka)

○	○	○	○	○	○	○	○
nichts oder fast nichts	ca. 1 Einheit	ca. 2 Einheiten	ca. 3 Einheiten	ca. 4 Einheiten	ca. 5 Einheiten	ca. 6 Einheiten	> 6 Einheiten

14. Hast Du, seitdem Du heute morgen aufgestanden bist Alkohol konsumiert?

☐ nein
☐ ja

15. Wie viel Alkohol konsumierst Du an einem für Dich durchschnittlichen Tag?
(Die folgenden Mengen entsprechen einer Alkoholeinheit: 0,5 l Bier, 0,2 l Wein, 8 cl Wodka)

○	○	○	○	○	○	○	○
nichts oder fast nichts	ca. 1 Einheit	ca. 2 Einheiten	ca. 3 Einheiten	ca. 4 Einheiten	ca. 5 Einheiten	ca. 6 Einheiten	> 6 Einheiten

16. Hast Du gestern Cannabis konsumiert?
(Wie alle Angaben anonymisiert. Auch bei Konsum wirst Du weiterhin vergütet!)

☐ nein
☐ ja

17. Hast Du, seitdem Du seitdem Du heute morgen aufgestanden bist Cannabis konsumiert?

☐ nein
☐ ja

18. Konsumierst Du an einem für Dich durchschnittlichen Tag Cannabis?

☐ nein
☐ ja

Recruiting

Derzeit sind wir auf der Suche nach volljährigen Probanden für unsere Studie zum Einfluss von Neurofeedback Training auf das Kurzzeitgedächtnis. Hierbei kommen die Probanden an fünf aufeinanderfolgenden Tagen zur gleichen Uhrzeit je eine Stunde ins Labor S-01.115 (Regina Mundi) um ihr Alphaaktivität zu erhöhen. Für das Training selbst wird EEG registriert und ein Programm bereitgestellt, welches die aktuelle Alphaaktivität widergibt. Welche Strategie dabei zur Erhöhung der Alphaaktivität verwendet wird, bleibt den Probanden selbst überlassen. Vor und nach den Trainingseinheiten wird jeweils ein Kurzzeitgedächtnistest durchgeführt und die gesamte Studie wird mit 6 Versuchspersonenstunden vergütet.

Informed Consent

Einverständniserklärung

Diese Studie beschäftigt sich mit der Frage, ob ein Training namens *Neurofeedback* mit kognitiver Leistung zusammenhängt.

Während des Trainings wird die kortikale Aktivität der Probanden mit Hilfe von EEG-Messung erfasst, aufgenommen und auf einem Bildschirm präsentiert. Das Training selbst besteht darin, die widergespiegelte kortikale Aktivität zu erhöhen.

Wir untersuchen diesen Zusammenhang um herauszufinden, ob Neurofeedback eine realistische Option für den Durchschnittsverbraucher wäre um seine oder ihre kognitiven Fähigkeiten zu steigern.

Die Studie wird von der *Arbeitsgruppe Kognitive Ergonomie und Arbeitspsychologie der Universität Fribourg* durchgeführt. Die Studie birgt keine größeren Risiken, als sie der Alltag mit sich bringt.

Die Untersuchung dauert üblicherweise 60 Minuten und ist vollständig anonymisiert (es sind keine Rückschlüsse auf teilnehmende Personen möglich). Im ersten Schritt wirst Du einen Digit Span Test durchführen und einige Fragen beantworten. Anschließend werden wir Deine Individuelle Alpha Aktivität erfassen. Dann lernst Du mit Hilfe eines Computerprogramms, wie Du Deine Alpha Aktivität erhöhen kannst. Danach absolvierst Du den Digit Span Test und beantwortest die Fragen ein zweites Mal.

Alle Antworten werden vertraulich behandelt und zu keinem Zeitpunkt sind Rückschlüsse auf einzelne Personen möglich. Alle Daten werden zusammengetragen und nur in aggregierter Form publiziert.

Psychologiestudierende der *Universität Fribourg*, welche an der Studie teilnehmen, erhalten dafür 6 Versuchspersonenstunden. Die Teilnahme ist freiwillig. Durch eine

Verweigerung der Teilnahme entstehen keine Nachteile für mögliche teilnehmende Personen und es wird nicht auf Vorteile verzichtet, auf die diese ansonsten Anspruch hätten. Die Teilnahme kann jederzeit ohne Angabe von Gründen und ohne darauffolgende Nachteile zurückgezogen werden.

Falls Teilnehmende weitere Fragen zu ihren Rechten haben oder sie eine Beschwerde oder Bedenken einreichen möchten, werden diese gebeten, sich beim Studienverantwortlichen Andreas Sonderegger zu melden.

Falls Du 18 Jahre oder älter bist, die aufgeführten Inhalte verstehst und Dich freiwillig bereit erklärst an der Studie teilzunehmen, klicke bitte auf "weiter", um die Untersuchung zu beginnen

Procedure from Participant's Point of View

Via and university intern E-Mail distribution list, Participants are informed about the possible to participate in the current study. Already in the first e-mail, we give an overview of the experiment (see "Recruiting", p. 61) and encourage interested persons to contact the experimenter. Afterwards we fixate the appointment and contact the participants again on the day before the appointment.

On the first test day, the experimenter welcomes the participant, before he or she reads and signs the informed consent. Afterwards, the EEG headset is attached and the study begins with a questionnaire about physical activity, substance intake (cannabis and alcohol) and sleep. All instruction are given by means of text within an online questionnaire. The online questionnaire leads the participants to a short term memory test (*Digit-Span Task*) is conducted. During the digit span test, digits appear one after another on the screen. The participants are advised to memorize them and type them in after the presentation has finished. Afterwards, two 2-min EEG epochs in an eyes-open and eyes closed resting state are conducted. Then, a written introduction into the connection between alpha and short-term memory performance is given. While participants are encouraged to think about and use personal alpha enhancement strategies during the following training, a list with mental strategies is provided as well. The neurofeedback training consists of five 3-min periods with 30s of break in-between. During the break, participants are advised to write down the mental strategy they used in the preceding period to enhance their alpha activity. Then, the EEG headset is removed and the short-term memory is conducted a second time. Sending-off the participant off, we remind him of the test session on the following day. The procedure during the test days 2 to 4 is identical except for one difference: only the most successful strategy is used during the five periods of

neurofeedback training. On the fifth and last test day, only one digit-span test is conducted and the credit points on a university intern reward system are granted. For the purpose of debriefing, participants are contacted via e-mail and informed about date and time of a debriefing presentation, during which information regarding the group (experimental or control) will be provided as well.

Online Questionnaire Instruction – Short-Term Memory Test

Nun wirst Du den Digit Span Test durchführen.

Dabei erscheinen Nummern auf dem Bildschirm. Diese sollst Du Dir merken.

Der Test beginnt mit einer dreistelligen Nummer.

Tippst Du sie korrekt ein folgt eine vierstellige Nummer.

So schreitet der Test fort, bis Du zwei Fehler hintereinander machst.

Bitte versuche nicht Deine Eingabe mit "←" oder "entf" zu korrigieren.

Viel Spaß!

Online Questionnaire Instruction – EEG Baseline Measurement

Nun werden wir Deine individuelle Alpha Aktivität bemessen.

Nachdem der Versuchsleiter Dir das EEG-Headset aufgesetzt hat, wirst Du Dich zwei Minuten lang mit geschlossen Augen entspannen.

Anschließend folgt eine weitere Entspannungsphase, wobei Du diesmal die Augen offen hast.

Da Muskelaktivität sowie Blinzeln das EEG-Signal stört, möchten wir Dich bitten die folgenden Punkte zu beachten:

- Nehme zu Beginn eine angenehme und entspannte Haltung ein, die es Dir ermöglicht Dich während der zwei Minuten so wenig wie möglich zu bewegen. Das Chin Rest zu Deiner Linken wird Dir dabei helfen.

- Versuche nicht zu blinzeln.

Der Versuchsleiter kommt jeden Moment herein.

Written Introduction into the Connection between Alpha Activity and Short-Term Memory Performance (first test day)

Die meisten Menschen haben ganz individuelle Strategien, um ihre Alpha Aktivität zu erhöhen und bei einer anderen Person hat die gleiche Strategie oft nicht den gleichen Effekt.

Hier trotzdem einige Beispiele/Themen, an welchen Du Dich orientieren kannst:

- Positive und negative Emotionen hervorrufen
- An Dinge denken, die mit Positivem verknüpft sind
- Körperliche Ruhe, ruhige Atmung
- Visualisieren verschiedener Tätigkeiten
- Liebe

Verwende beim nun folgenden Neurofeedback Training bei jeder Einheit nur eine Strategie und verwende jede Strategie nur einmal im Laufe der 5 Einheiten.

Bitte notiere in jeder der Pausen die verwendete Strategie stichwortartig auf dem dafür vorgesehenen Blatt.

Versuch während den Pausen bitte nicht Deine Alpha Aktivität zu erhöhen, sondern denke an etwas anderes.

Viel Spass!

Written Instruction before Neurofeedback Training (test days 2 to 4)

Der Versuchsleiter hat für Dich analysiert, welches die für Dich beste Strategie ist um Deine individuelle Alpha Aktivität zu erhöhen. Er wird sie Dir gleich mitteilen.

Bitte verwende von nun an nur noch diese Strategie um Dein Alpha zu erhöhen.

Versuch während den Pausen bitte nicht Deine Alpha Aktivität zu erhöhen, sondern denke an etwas anderes.

Viel Spass!

Online Questionnaire Instruction – Neurofeedback Training (all test days)

Gleich öffnet sich das Alpha Trainingsprogramm.

Die Farbe am Hinterkopf des Display-Kopfes stellt Deine individuelle Alpha Aktivität dar.

Versuche sie so stark und lange wie möglich zu erhöhen, das heißt, erzeuge so viel rote Aktivität wie möglich.

Du wirst 5 Trainingseinheiten á drei Minuten durchführen.

Zwischen den Einheiten wirst Du je eine Pause von 30 Sekunden haben.

Wenn der Versuchsleiter den Raum verlässt beginnt die erste Einheit Deines Trainings. Klopft er das nächste Mal beginnt die Pause. Klopft er ein weiteres Mal beginnt die Zweite Trainingseinheit Deines Trainings usw.
